# Prevalence and discriminant validity of PTSD and CPTSD in a community sample of adolescents with refugee backgrounds residing in Sweden

**DOI:** 10.1007/s00787-025-02858-8

**Published:** 2025-10-08

**Authors:** Johan Andersson, Carolina Bråhn, Hongru Zhai, Erica Mattelin, Ann-Charlotte Münger,, Laura Korhonen

**Affiliations:** 1 Barnafrid and Department of Biomedical and Clinical Sciences, Linköping University, Linköping, Sweden; 2 Save the Children, Sweden; 3 Center for Social and Affective Neuroscience and Department of Biomedical and Clinical Sciences, Linköping University, Linköping, Sweden; 4 Clinical Department of Child and Adolescent Psychiatry in Linköping, Region Östergötland, Linköping, Sweden

**Keywords:** Refugees, Adolescent, PTSD, CPTSD, Latent variable modeling

## Abstract

Research on complex posttraumatic stress disorder (CPTSD) among individuals with refugee backgrounds is limited, and its validity within this group remains underexplored. This study aimed to assess the prevalence and discriminant validity of posttraumatic stress disorder (PTSD) and CPTSD, as well as the risk factors for CPTSD, in a community sample of adolescents with refugee backgrounds residing in Sweden. This study involved 296 adolescents. Probable diagnoses were evaluated according to DSM-5 and ICD-11 criteria. Latent class analysis was employed to examine the discriminant validity of PTSD and CPTSD, while logistic regression analysis was used to explore risk factors for CPTSD. The findings indicated that 24.1% had a probable diagnosis of PTSD according to the DSM-5. For ICD-11, the equivalent proportions were 7.1% for PTSD and 10.8% for CPTSD. Latent class analysis identified three distinct classes: Low symptoms (46.9%), PTSD (29.6%), and CPTSD (23.6%). Compared to the PTSD class, membership in the CPTSD class was predicted by exposure to more types of violence and child maltreatment. It was also associated with higher posttraumatic stress symptoms, worse general functioning, poorer mental well-being, increased suicidal thoughts, more treatment-seeking behavior, and greater comorbidity. This study found a high prevalence of PTSD and CPTSD among adolescents with refugee backgrounds living in Sweden. Distinct classes aligned with the ICD-11 formulation of PTSD and CPTSD were identified, with exposure to violence and child maltreatment emerging as key risk factors for CPTSD. Results underscore the importance of identifying and addressing posttraumatic stress in adolescents with refugee backgrounds. Future research should aim to further validate the CPTSD diagnosis in larger samples of adolescents with refugee backgrounds.

**Supplementary Information:**

The online version contains supplementary material available at 10.1007/s00787-025-02858-8.

## Introduction

Complex posttraumatic stress disorder (CPTSD) was introduced in the 11th version of the International Classification of Diseases (ICD-11) as a ‘sibling’ diagnosis to PTSD, sharing core elements while adding symptoms related to disturbances of self-organization (DSO) [1]. It was first theorized by Hermann [2] as the typical presentation of symptoms following repeated, prolonged, and interpersonal potentially traumatic events (PTEs). Although not incorporated into the diagnostic criteria and instead regarded as a risk factor for the diagnosis [3], evidence is mounting that these categories of exposures are more prevalent in individuals diagnosed with CPTSD [4–6]. Since these types of exposures, such as repeated physical and sexual abuse and human trafficking [7], are more common in people with refugee backgrounds [8, 9], CPTSD may be particularly relevant for this group, as noted in previous literature [10, 11]. Furthermore, prevalence rates of PTSD in adolescents with refugee backgrounds are high compared to general populations [12, 13], further strengthening the relevance of studying the CPTSD diagnosis in this group.

Among adolescents without refugee backgrounds, factors such as interpersonal PTEs [5, 14], childhood PTEs [15], emotional abuse [6, 16], physical abuse in the family [17], neglect [6], and war experiences [18] have been associated with an increased risk of developing CPTSD. In adults with refugee backgrounds, interpersonal PTEs [11, 19], visa insecurity [20], reception conditions [21], and early age of first onset of a PTE [21] have similarly been linked to higher CPTSD risk. Studies have also identified a dose–response effect between this population's cumulative PTE exposure and CPTSD [19, 20]. However, research into the prevalence of and risk factors associated with CPTSD among individuals with refugee backgrounds remains limited [22], particularly concerning children, where, to our knowledge, no research currently exists.

According to ICD-11, a person is diagnosed with CPTSD if they have experienced an extremely threatening or horrific event and fulfill the criteria for at least one of two symptoms in each of the three symptom clusters included in PTSD: re-experiencing of the traumatic event(s) (Re), avoidance of reminders likely to produce re-experiencing of the traumatic event(s) (Av), and a persistent sense of threat (Th). Additionally, an individual must fulfill the criteria for at least one of two symptoms in each of the three symptom clusters within the DSO category, which include affective dysregulation (AD), negative self-concept (NSC), and disturbed relations (DR). Furthermore, the symptoms must have persisted for several weeks and have caused functional impairment. CPTSD was not included as a diagnosis in the fifth edition of the Diagnostic and Statistical Manual of Mental Disorders (DSM-5) [23]. In DSM-5, PTSD is more broadly defined and characterized by 20 symptoms across four distinct symptom clusters: intrusion (In), avoidance (Av), negative alterations in cognitions and mood (NACM), and alterations in arousal and reactivity (AAR). To be diagnosed with PTSD, an individual must fulfill the criteria for at least one symptom in the In and Av clusters and at least two symptoms in the NACM and AAR clusters. Additionally, symptoms need to have persisted for more than a month and cause significant clinical suffering and/or functional impairment.

Since its inclusion in the ICD-11, the validity of CPTSD has been debated, yet evidence supports its factorial and discriminant validity. Studies that employ confirmatory factor analysis have frequently identified a correlated six-factor model or a two-factor higher-order model, both consistent with ICD-11 PTSD/CPTSD. Conversely, studies utilizing mixture models (e.g., latent class analysis (LCA)) have consistently identified distinct classes representative of the ICD-11 PTSD/CPTSD formulation [24]. Research has included both adults [25] and children [17] and has been conducted in various cultural contexts with clinical [26] and non-clinical samples [27]. Nonetheless, additional studies on high-trauma populations, including children and adolescents, are needed [24]. To date, 14 studies have examined factorial and discriminant validity in refugee samples (e.g. [10, 19, 20, 28], see Online Resource 1 for the complete list). In LCA studies conducted among populations with refugee backgrounds, the membership in a CPTSD class has been associated with elevated levels of functional impairment [11, 21, 29], higher levels of depressive symptoms [30], and lower quality of life [30]. However, it is noteworthy that no validation study on CPTSD involving individuals with refugee backgrounds has included children in their samples, and only one has focused on young adults [17].

### Aim and hypotheses

This study aimed to present prevalence figures for PTSD and CPTSD in a community sample of adolescents with refugee backgrounds living in Sweden. Furthermore, the study sought to examine the discriminant validity of PTSD and CPTSD, along with potential risk factors for CPTSD in this group. We hypothesized that:

1. The rates of PTSD and CPTSD would be higher compared to community samples of individuals without refugee backgrounds.
2. Latent classes based on the ICD-11 conceptualization of PTSD and CPTSD would be identified in the sample, along with a low symptomatic class.
3. Membership in the CPTSD class could be predicted by covariates like exposure to violence.

## Methods

### Participants

This study is part of the research project ‘Long Journey to Shelter’. The published protocol [31] and the primary study of the larger research project [32] provide additional information about the project, participants, and procedures. The Regional Ethics Board of Linköping (2018/292–31 and 2018/504–32) and the National Ethics Board (2019–05473, 2020–00949, and 2021–03001) granted ethical approval.

The participants included 296 adolescents, aged 12 to 25 years, with refugee backgrounds residing in Sweden. In this study, we defined ‘adolescence’ as the period of life spanning from 10 to 24 years, following contemporary definitions that consider important biological and psychological growth in this period [33]. The chosen age range also catches the critical transition period from child to adult mental health services. Of the sample, 161 participants were children aged 12 to 18, and 135 were young adults aged 18 to 25. The groups overlapped in age because some participants in the 12–18 age group reached the age of 18 between consenting to participate and the assessment.

### Procedure

Participants were recruited through social services, schools, healthcare services, social media, and non-governmental organizations from 2019 to 2022. Professionals directly working with the target group asked potential participants or their legal guardians about their interest in participating. All information about the study was provided both verbally and in writing in the participants' preferred language, and written consent was obtained from all participants. Structured quantitative interviews were conducted in the participants’ preferred language, mostly with interpreter assistance. An Arabic-speaking research assistant conducted most of the Arabic interviews. Participants chose the interview locations, but most were conducted via Zoom due to COVID-19 restrictions.

### Measures

#### PTSD/CPTSD

To determine a probable PTSD and CPTSD diagnosis and assess posttraumatic stress symptoms (PTSS), two different questionnaires were utilized depending on the age of the participant. The Child and Adolescent Trauma Screen version 1 (CATS-1) [34], a 20-item scale based on the DSM-5 criteria for PTSD, was utilized for children. Symptom items were rated on a 4-point scale (0 – Never, 1 – Once in a while, 2 – Half the time, 3 – Almost always). Five additional items indicated functional impairment in different life domains (yes/no). For young adults, the 20-item Posttraumatic Stress Disorder Checklist for DSM-5 (PCL-5) [35] was utilized instead. Symptom items were rated on a 5-point scale (0 – Not at all, 1 – A little bit, 2 – Moderately, 3 – Quite a bit, 4 – Extremely). The PCL-5 did not include items related to functional impairment. For both the CATS-1 and the PCL-5, each symptom item rated ≥ 2 was considered a symptom endorsed for diagnostic purposes. Both questionnaires were developed according to the DSM-5 conceptualization of PTSD. For use with ICD-11, items were selected to align with ICD-11 criteria for PTSD and CPTSD. Item selection corresponding to ICD-11 criteria can be found in Table S1 in Online Resource 1.

#### Covariates

Interview questions collected information on age, gender (male/female/other), country of origin, parental education, unaccompanied status, and whether participants had ever sought care for a mental health problem (yes/no). Information about transforming variables for regression analysis can be found in Online Resource 1.

Data on violence was collected using the Juvenile Victimization Questionnaire (JVQ) [36], a 34-item questionnaire constructed to capture experiences of various types of violence. This study used child maltreatment, consisting of four items, and sexual victimization, consisting of six items, as violence categories in the analysis. The 5-item World Health Organization Well-Being Index (WHO-5) [37] measures mental well-being using a 6-point scale (5—All of the time, 4—Most of the time, 3—More than half the time, 2—Less than half the time, 1—Some of the time, 0—At no time). General functioning was assessed using the clinician-rated Global Assessment of Functioning (GAF) [23] for young adults and the Children’s Global Assessment Scale (CGAS) [38] for children. Both scales rate general functioning from 1–100, with one representing the most impaired functioning. Two structured diagnostic interviews, the Mini International Neuropsychiatric Interview for Children and Adolescents 6.0 (MINI-KID) [39] and the Mini International Neuropsychiatric Interview 7.0 (MINI) [40], were employed to assess common mental disorders within the sample. One item from the Adolescent Resilience Questionnaire (ARQ) [41] was selected for diagnosing probable CPTSD. More details on the questionnaires can be found in Online Resource 1.

#### Reliability

Internal consistency for CATS-1 (α = 0.94), PCL-5 (α = 0.90), and WHO-5 (α = 0.86) were strong in the sample. Additional details on the measures can be found in Online Resource 1.

### Data analysis

A probable diagnosis of PTSD and CPTSD was established by adhering to the diagnostic criteria outlined in the DSM-5 and ICD-11. All participants had to have experienced a potentially traumatic event, as indicated by the JVQ. Regarding symptom endorsement, for DSM-5 PTSD, this required at least one symptom from clusters In and Av, and a minimum of two symptoms from clusters NACM and AAR. For ICD-11 PTSD, it necessitated at least one symptom from clusters Re, Av, and Th. For CPTSD, at least one symptom from clusters AD, NSC, and DR was necessary, along with a probable PTSD diagnosis. Additionally, the endorsement of any functional impairment was required for participants who completed CATS-1. Functional impairment was not assessed for participants who completed the PCL-5. Differences in diagnostic rates between DSM-5 and ICD-11 were analyzed using McNemar’s test. Gender and age group differences in diagnostic rates for all probable diagnoses were examined using Fisher’s exact test. Furthermore, significant differences in PTSS scores between the ICD-11 PTSD group and the ICD-11 CPTSD group were evaluated using t-tests.

LCA was utilized to evaluate the discriminant validity of PTSD and CPTSD within the sample. ICD-11 symptom clusters extracted from the CATS-1 and the PCL-5 served as indicators for classes. The clusters were treated as categorical variables, with a rating of ≥ 2 on at least one of the included symptoms indicating cluster endorsement. Participants who did not complete these questionnaires were excluded from the LCA, resulting in a sample of 258 participants.

LCA was conducted using Mplus Version 8 [42]. Missing data on CATS-1 and PCL-5 items was addressed through mean imputation. Two participants had missing data on all the CATS-1 items but had completed the questionnaire. In these cases, missing data was imputed using the MissForest random forest imputation algorithm [43]. Missing data was 0.4% on PCL-5 and 2.6% on CATS-1. The regression analysis was performed with complete case data. The proportion of missing data on the covariates included in the regression analysis ranged from 0% to 16.9%.

The optimal number of classes was determined using established fit indices, by inspecting plots, and assessing theoretical meaningfulness. The Bayesian information criterion (BIC) [44], the sample size-adjusted Bayesian information criterion (SABIC) [45], and Akaike’s information criterion (AIC) [46] were examined for fit. Significance on the bootstrapped likelihood ratio test (BLRT) [47] was also sought, along with high entropy and reasonable class sizes. LCA was conducted with 500 random starts, 50 first-stage iterations, and 100 final optimizations to ensure the replication of the highest log-likelihood value. We tested 1 to k classes, stopping when adding more classes worsened the fit Table 1.

A logistic regression analysis was performed to further investigate the discriminant validity of PTSD and CPTSD and examine risk factors for CPTSD. We ran an unadjusted model followed by an adjusted model including confounders. The PTSD class was set as the reference class for all analyses. The Likelihood ratio test (LRT) was used to assess model fit and McFadden’s pseudo R^2^ [48] as a measure of explained variance. The regression analysis was run in R using the nnet package [49].

## Results

### Sample characteristics

Table 2 summarizes sociodemographic information. The total sample consisted of 296 participants, of whom 45.3% were female, and the mean age was 17.98 years.

**Table 1:** Sociodemographic characteristics of the sample

Variable	Total (N = 296)	Children (N = 161)	Young adults (N = 135)
Age, years, mean (SD^a^) Min/max Missing	17.98 (3.75) 12/25 0	15.12 (1.86) 12/18 0	21.39 (2.29) 18/25 0
Gender, female, n (%) Missing	134 (45.3%) 0	73 (45.3%) 0	61 (45.2%) 0
Region of origin, n (%): Middle East & North Africa Sub-Saharan Africa Other Missing	210 (71.2%) 78 (26.4%) 7 (2.4%) 1	80 (49.7%) 74 (46.0%) 7 (4.4%) 0	130 (97.0%) 4 (3.0%) 0 1
Asylum status, n (%): Decision received Awaiting decision Appealing Do not know Missing	223 (75.9%) 44 (15.0%) 5 (1.7%) 22 (7.5%) 2	95 (59.7%) 38 (23.9%) 4 (2.5%) 22 (13.8%) 2	128 (94.8%) 6 (4.5%) 1 (0.7%) 0 0
Years in Sweden, mean (SD) Min/max Missing, n	2.54 (2.89) 0.08/13.42 2	0.31 (0.14) 0.08/1 1	5.20 (2.29) 0.08/13.42 1
Unaccompanied, n (%) Missing	70 (23.7%) 0	26 (16.1%) 0	44 (32.6%) 0
Parental education, (%) High Medium Low Missing	99 (35.2%) 52 (18.5%) 130 (46.3%) 15	22 (14.5%) 27 (17.8%) 103 (67.8%) 9	77 (59.7%) 25 (19.4%) 27 (20.9%) 6

^a^ Standard deviation

### Prevalence rates of PTSD and CPTSD

**Fig. 1:**
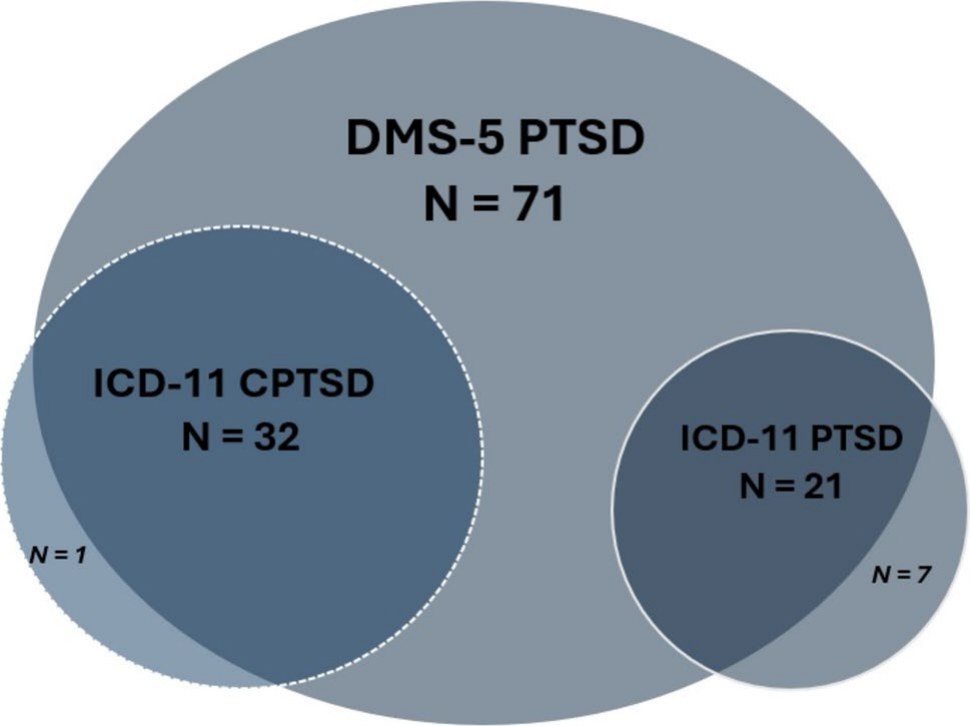
Venn diagram of distribution of probable diagnoses according to DSM-5 and ICD-11

Table 2 provides prevalence rates of PTSD and CPTSD in the sample and Fig 1 illustrates the distribution of probable diagnoses according to the two diagnostic systems. In the total sample, 24.1% had a probable diagnosis of PTSD according to the DSM-5. For the ICD-11, the corresponding proportions were 7.1% for PTSD and 10.8% for CPTSD. Prevalence was higher among young adults for DSM-5 PTSD (Fischer’s test; *p* = < 0.01), ICD-11 PTSD (Fischer’s test; p = 0.02), and ICD-11 CPTSD (Fischer’s test; *p* = < 0.01).

**Table 2:** Probable diagnoses according to DSM-5 and ICD-11 criteria

Diagnosis	Total sample (N = 296)	Children (N = 161)	Young adults (N = 135)
DSM-5 PTSD, n (%)	71 (24.1%)	15 (9.4%)	56 (41.5%)
ICD-11 PTSD, n (%)	21 (7.1%)	6 (3.8%)	15 (11.1%)
ICD-11 CPTSD, n (%)	32 (10.8%)	5 (3.1%)	27 (20.0%)
Missing	1	1	0

Significance testing showed significant differences in diagnostic rates between DSM-5 PTSD and ICD-11 PTSD + CPTSD (McNemar’s test; *p* = < 0.01). No significant gender differences were found in diagnostic rates for DSM-5 PTSD (Fischer’s test; *p* = 0.79), ICD-11 PTSD (Fischer’s test; *p* = 1), or ICD-11 CPTSD (Fischer’s test; *p* = 0.19). However, PTSS scores were significantly higher in the ICD-11 CPTSD group (M = 1.34, SD = 0.71)^1^ compared to the ICD-11 PTSD group (M = 0.76, SD = 0.82),^1^ (t(38.53) = 2.65, p = 0.01).

### Latent class analysis

Table 3 presents fit indices for one to four classes. The highest log-likelihood value was replicated in all solutions. The BLRT was no longer significant when a fourth class was added; therefore, further analysis focused on solutions with one to three classes. AIC and SABIC favored the three-class solution, while BIC and entropy indicated a preference for the two-class solution.

**Table 3:** Fit indices for 1 to 5 classes

Classes	logLik	AIC	BIC	SABIC	BLRT (*p)*	Entropy
1	−1040.5	2093.1	2114.4	2095.4		1.000
2	−829.0	1683.9	1730.1	1688.9	< 0.000	0.860
**3**	**−818.5**	**1677.0**	**1748.0**	**1684.6**	** < 0.000**	**0.726**
4	−811.1	1676.1	1772.0	1686.4	0.109	0.776
5	−805.8	1679.6	1800.4	1692.6	1.000	0.811

**Fig. 2:**
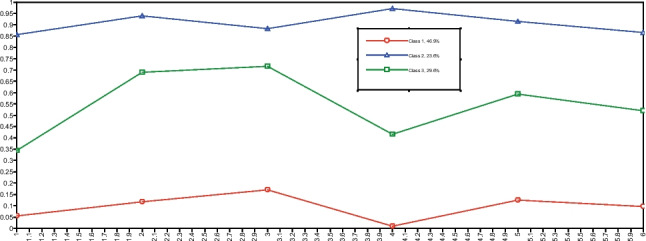
Profile plot for the three-class solution

Analysis of plots revealed that the two-class solution reflected levels of symptom severity, while the three-class solution provided distinct classes that differed not only in severity but also in their endorsement of various symptom clusters. Furthermore, the three-class solution aligned with the hypothesis of PTSD, CPTSD, and a low-symptom class, and was, therefore, chosen. The profile plot is presented in Fig. 2. Figure S1 in Online Resource 1 shows profile plots for the two-class solution.

Class 1, characterized by generally low endorsement of symptom clusters, was named the Low Symptoms class and comprised 46.9% of the sample. Class 2, marked by high endorsement of both PTSD symptom clusters and DSO clusters, was designated the CPTSD class and accounted for 23.6% of the sample. Class 3 displayed moderate to low endorsement of the Re cluster, high endorsement of the Av and Th clusters, and moderate endorsement of the DSO clusters. This class was identified as the PTSD class and made up 29.6% of the sample. Table S2 in Online Resource 1 shows the symptom cluster endorsement percentages for each class.

### Logistic regression analysis

Table 4 provides descriptive statistics for the included covariates by class and the total sample in the LCA. The distinction between the PTSD and CPTSD classes was explained by the unadjusted logistic regression analysis.

**Table 4:** Descriptives of covariates of classes and differences between CPTSD and PTSD based on unadjusted regression model

Covariates	Total LCA sample (n = 258)	Class 1 Low symptoms (n = 122)	Class 2 CPTSD (n = 65)	Class 3 PTSD (n = 71)	Significant differences between CPTSD and PTSD (*p* < 0.05)	LRT *p*	McFadden’s R^2^
Age, years, mean (SD) Min/max Missing	18.21 (3.75) 12/25 0	16.33 (3.17) 12/25 0	19.98 (3.33) 12/25 0	19.83 (3.50) 12/25 0		< 0.01	0.12
Gender, female, n (%) Missing	115 (44.6%) 0	58 (47.5%) 0	26 (40.0%) 0	31 (43.7%) 0		0.60	< 0.01
Unaccompanied status, n (%) Missing	61 (23.6%) 0	11 (9.0%) 0	29 (44.6%) 0	21 (29.6%) 0		< 0.01	0.06
Exposure to number of different types of violence, mean (SD) Min/max Missing	10.97 (7.22) 0/34 1	7.33 (6.03) 0/25 1	15.78 (7.10) 4/34 0	12.76 (5.88) 2/25 0	CPTSD > PTSD (*z* = 2.47)	< 0.01	0.13
Exposure to sexual victimization, yes, n (%) Missing	66 (25.8%) 2	15 (12.4%) 1	29 (44.6%) 0	22 (31.4%) 1		< 0.01	0.05
Exposure to child maltreatment, yes, n (%) Missing	120 (46.9%) 2	34 (28.1%) 1	47 (72.3%) 0	39 (55.7%) 1	CPTSD > PTSD (*z* = 1.99)	< 0.01	0.07
CATS-1, mean (SD) Min/max Missing	11.77 (13.07) 0/54 0	5.20 (5.70) 0/19 0	38.23 (7.03) 28.50/54 0	22.58 (8.39) 8/36 0	See PTSS	-	-
PCL-5, mean (SD) Min/max Missing	29.91 (17.09) 0/69 0	10.84 (6.05) 0/23 0	45.69 (11.30) 22/69 0	24.43 (10.90) 8/57.89 0	See PTSS	-	-
PTSS, mean^a^ (SD) Min/max Missing	See CATS-1 and PCL-5	−0.82 (0.49) −1.75/0.55 0	1.18 (0.79) −0.46/3.23 0	0.02 (0.83) −1.28/1.85 0	CPTSD > PTSD (*z* = 5.98)	< 0.01	0.33
GAF/C-GAS, mean (SD) Median Min/max Missing	80.65 (14.64) 85 31/100 4	86.63 (9.47) 90 55/100 3	68.08 (15.70) 69.50 35/98 1	81.96 (13.90) 86 31/97 0	CPTSD < PTSD (*z* = −4.72)	< 0.01	0.13
WHO-5, mean (SD) Median Min/max Missing	69.56 (24.59) 72 0/100 9	80.58 (20.50) 88 20/100 5	52.00 (25.40) 52 0/100 4	65.97 (20.60) 68 12/100 0	CPTSD < PTSD (*z* = −3.00)	< 0.01	0.11
Suicide thoughts, yes, n (%) Missing	33 (14.2%) 26	6 (5.9%) 20	19 (30.6%) 3	8 (11.8%) 3	CPTSD > PTSD (*z* = 2.57)	< 0.01	0.04
Any other diagnosis, yes, n (%) Missing	71 (28.1%) 5	16 (13.4%) 3	40 (62.5%) 1	15 (21.4%) 1	CPTSD > PTSD (*z* = 4.65)	< 0.01	0.09
Sought treatment, yes, n (%) Missing	57 (22.7%) 7	13 (10.9%) 3	28 (45.2%) 3	16 (22.9%) 1	CPTSD > PTSD (z = 2.67)	< 0.01	0.05

Notes: LRT = Likelihood Ratio Test

^a^ Standardized scores

Table 5 presents the results of the adjusted logistic regression analysis. Differences are presented as odds ratios (OR) or adjusted odds ratios (aOR) along with 95% confidence intervals (CI).

**Table 5:** Differences between CPTSD and PTSD based on adjusted logistic regression analysis with PTSD as the reference class

Variable	Confounders	*p*	Wald Z	aOR	95% CI	LRT *p*	McFadden’s R^2^
Age		0.80	0.26	1.01	[0.14, 1.26]	< 0.01	0.12
Gender		0.67	0.43	1.16	[0.59, 2.30]	0.60	< 0.01
Unaccompanied status	Age, Gender	0.08	1.75	1.97	[0.92, 4.23]	< 0.01	0.15
**Exposure to number of different types of violence**	Age, Unaccompanied status	**0.03**	**2.14**	**1.06**	**[1.01, 1.12]**	** < 0.01**	**0.19**
Exposure to sexual victimization	Age, Gender, Unaccompanied status	0.17	1.36	1.68	[0.80, 3.52]		
**Exposure to child maltreatment**	Age	**0.047**	**1.98**	**2.08**	**[1.01, 4.30]**	** < 0.01**	**0.16**
**PTSS**^**a**^	Gender, Unaccompanied status, Exposure to violence, Exposure to child maltreatment, Exposure to sexual victimization	** < 0.01**	**5.70**	**5.50**	**[3.06, 9.88]**	** < 0.01**	**0.38**
**GAF/C-GAS**	Age, Gender, Unaccompanied status, Exposure to violence, Exposure to child maltreatment, Exposure to sexual victimization	** < 0.01**	**−4.48**	**0.93**	**[0.91, 0.96]**	** < 0.01**	**0.29**
**WHO-5**	Age, Gender, Unaccompanied status, Exposure to violence, Exposure to child maltreatment, Exposure to sexual victimization	**0.01**	**−2.55**	**0.98**	**[0.96, 0.995]**	** < 0.01**	**0.24**
**Suicide thoughts**	Age, Gender, Unaccompanied status, Exposure to violence, Exposure to child maltreatment, Exposure to sexual victimization	**0.02**	**2.29**	**3.27**	**[1.19, 8.99]**	** < 0.01**	**0.21**
**Any other diagnosis**	Age, Gender, Unaccompanied status, Exposure to violence, Exposure to child maltreatment, Exposure to sexual victimization	** < 0.01**	**4.30**	**6.05**	**[2.66, 13.70]**	** < 0.01**	**0.25**
**Sought treatment**	Age, Gender, Unaccompanied status, Exposure to violence, Exposure to child maltreatment, Exposure to sexual victimization	**0.04**	**2.04**	**2.40**	**[1.04, 5.56]**	** < 0.01**	**0.21**

Notes: LRT = Likelihood Ratio Test

^a^ Standardized scores

No significant differences were identified between the CPTSD and PTSD classes regarding age, gender, unaccompanied status, and exposure to sexual victimization. Participants in the CPTSD class were significantly more likely to have experienced a greater number of different types of violence (aOR 1.06, CI 1.01–1.12) and to have been subjected to child maltreatment to a greater extent (aOR 2.08, CI 1.01–4.30) compared to those in the PTSD class. Belonging to the CPTSD class was also associated with higher PTSS scores (aOR 5.50, CI 3.06–9.88), lower general functioning (aOR 0.93, CI 0.91–0.96), poorer mental well-being (aOR 0.98, CI 0.96–0.995), increased comorbidity (aOR 6.05, CI 2.66–13.70), and more treatment-seeking behavior (aOR 2.40, CI 1.04–5.56) compared to membership in the PTSD class. Additionally, participants in the CPTSD class exhibited a higher prevalence of suicidal thoughts (aOR 3.27, CI 1.19–8.99) compared to those in the PTSD class.

## Discussion

To our knowledge, this is the first study to investigate the discriminant validity of PTSD and CPTSD in a sample that includes children with refugee backgrounds and one of the few that also accounts for young adults. It also provides estimates of the prevalence rates of CPTSD among adolescents with refugee backgrounds.

Per our second and third hypotheses, the study identified a low-symptom class, a PTSD class, and a CPTSD class, and found that exposure to more types of violence and child maltreatment predicted CPTSD class membership. Significant differences in PTSS scores, general functioning, mental well-being, suicidal thoughts, comorbidity, and treatment-seeking behavior further validated the distinction between diagnoses.

The total sample demonstrated a probable diagnostic rate of 24.1% for DSM-5 PTSD, while the combined rate for ICD-11 PTSD and CPTSD was 17.9%. These prevalence rates are high compared to diagnostic estimates for general populations in high-income countries, where studies have reported a lifetime prevalence of PTSD at 3.9% for adults [50]. Compared to global estimates for refugee populations, the prevalence rates align more closely with those estimated by systematic reviews (22.71% for children [13] and 31.46% for adults [12]). These findings further substantiate the high burden of PTSD in populations with refugee backgrounds. The differences compared to general populations are likely due in part to the high exposure to violence and other PTEs [8, 9], but they may also reflect other prevalent difficulties known to be associated with mental health outcomes, such as post-migration stress [51]. However, the rates in this study are lower than those reported in Swedish studies examining PTSD prevalence in minors with refugee backgrounds, where estimates have ranged from 34 to 76% [52–54]. The probable prevalence of CPTSD was 10.8% in the total sample, compared to 2.2–9.3% in a systematic review assessing prevalence rates in adult populations with refugee backgrounds [22].

While the results are not necessarily generalizable to all adolescents with refugee backgrounds residing in Sweden, the prevalence rates in this study were lower than those reported in previous Swedish studies. These differences might partly be explained by the use of different questionnaires for assessment. The present study opted for the more extensive CATS-1 and PCL-5 questionnaires, while the previous studies all used the shorter Children’s Revised Impact of Event Scale (8-item or 13-item version) [55]. Furthermore, the present study consistently used trained interpreters or researchers who were fluent in the participants' preferred language and conducted assessments in interview form, a departure from the methodologies employed in the other studies mentioned. Nevertheless, the rates remain high, underscoring the importance of identifying and addressing posttraumatic stress in adolescents with refugee backgrounds. For participants with a probable ICD-11 CPTSD diagnosis, this may be particularly relevant, as they reported a significantly higher symptom burden, worse general functioning, poorer well-being, and more suicidal thoughts compared to those with a probable ICD-11 PTSD diagnosis. Effective and evidence-based trauma treatment options for children and adults, such as trauma focused cognitive behavioral therapy [56, 57], prolonged exposure [58], and eye movement desensitization and reprocessing [57, 58], are available but previous Swedish research indicates that there are barriers to accessing mental health care for adolescents with refugee backgrounds that must be addressed [59]. Referral agents, such as professionals in social services, primary care, and schools, must be aware of the burden facing adolescents with refugee backgrounds and initiate contact and collaboration with mental health services as needed. Likewise, an increased awareness of the heightened risk of CPTSD among professionals within mental health services is crucial for identification and access to treatment. Additionally, there is a need for further research regarding treatment options specifically designed to target CPTSD [60] and a pressing need to develop clinical guidelines for the diagnosis [61].

Young adults exhibited higher probable rates of PTSD and CPTSD compared to children. This phenomenon may be indicative of a dose–effect relationship between exposure to violence and PTSD/CPTSD. Additionally, it may indicate that coping with violence varies according to one's developmental phase, which is a natural part of the maturation process during adolescence.

The ICD-11 diagnostic rates were significantly lower than those of the DSM-5. This is likely due to the relatively low endorsement of the ICD-11 re-experiencing cluster (see Table S2 in Online Resource 1). This was also evident in the LCA, where the PTSD group showed a surprisingly low re-experiencing cluster endorsement (33.8%). The lower diagnostic rates for ICD-11 have been demonstrated previously; for example, Hyland et al. [62] have argued that ICD-11’s re-experiencing cluster may be too narrowly defined to capture a sufficient number of individuals with clinically meaningful levels of distress. As a sensitivity analysis, we conducted an additional LCA, adding an item from the CATS-1 and the PCL-5 relating to re-experiencing upsetting thoughts and images to the re-experiencing cluster. The results showed increased re-experiencing cluster endorsement in the PTSD class while maintaining the three-class solution and participant distribution between classes (see Figure S2 in Online Resource 1). Endorsement of the re-experiencing cluster in the CPTSD class was high in both analyses, which may indicate that the narrow definition of the re-experiencing cluster in ICD-11 impacts this diagnostic group less.

The LCA yielded a three-class solution: a low-symptoms class and two classes consistent with the ICD-11 PTSD and CPTSD formulations. The entropy value (0.726) was on the low side for the selected solution, indicating some overlap between the classes. This might explain the differences between the PTSD and CPTSD class memberships and the probable diagnostic rates. Nevertheless, these results align with previous LCA studies on adolescents without refugee backgrounds (e.g. [17, 18]) and studies on adult samples with refugee backgrounds (e.g. [11, 29]). Although LCA studies used in this context generally identify a PTSD and a CPTSD class, the number of additional classes differs between studies. Sample size affects the ability to detect smaller classes, while the kind of sample (e.g., clinical, community-based, high or low exposure to violence) influences which types of classes can be identified. For instance, this study did not identify a DSO class, whereas previous studies on larger community samples have [15, 17]. These findings highlight the need for further validation of the diagnosis in more diverse and larger samples of adolescents with refugee backgrounds.

The relatively high endorsement of the DSO clusters in the PTSD class in this study may be attributable to the high exposure to violence seen in the sample (a mean of 10.97 types of violence). Membership in the CPTSD class was predicted by exposure to more types of violence and exposure to child maltreatment. The distinction between the diagnoses was further validated by significantly higher PTSS scores, lower general functioning, poorer self-rated mental well-being, increased suicidal thoughts, more treatment-seeking behavior, and greater comorbidity among participants in the CPTSD class. These findings highlight the severity of the CPTSD diagnosis and emphasize the need for clinical guidelines to assess and treat the condition, especially as the implementation of ICD-11 continues globally. Surprisingly, despite previous studies reporting such an association [17, 23] and given the interpersonal nature of this form of PTE, exposure to sexual victimization was not associated with CPTSD class membership in the present study. This may be due to limited statistical power, and further investigation is warranted.

The study results contribute to the growing body of evidence supporting the discriminant validity of the PTSD and CPTSD diagnoses and confirm previous research identifying childhood maltreatment as a risk factor for the development of CPTSD in adolescents [6, 16, 17], as well as illustrating a dose–response relationship between exposure to violence and CPTSD in populations with refugee backgrounds [19, 20]. Furthermore, the interpersonal nature of child maltreatment and the dose–response effect resonate with Herman’s original theory, suggesting that specific types of PTEs (interpersonal, repeated, and prolonged) underpin the diagnosis [2].

### Strengths and limitations

This study is not without limitations. The sample is not representative and relatively small for person-centered statistical analysis. This affects generalizability and hinders analysis of reliability through split samples. The data collection was conducted from 2019 to 2022, with participants arriving up to 13.4 years earlier, thus limiting the findings to groups arriving as refugees in this period. Furthermore, the study utilized questionnaires not validated for the screening of ICD-11 PTSD and CPTSD, but only for DSM-5 PTSD, and added an item from a questionnaire designed to measure resilience (ARQ). Using a questionnaire designed for measuring ICD-11 PTSD/CPTSD would have been advantageous, but no such questionnaire was available when the study was planned. However, previous studies have employed a similar approach [20, 26, 28]. Another limitation is the absence of items on the PCL-5 addressing functional impairment, meaning that the estimated rates for young adults may be slightly inflated. Additionally, most questionnaires utilized in this study have not been extensively validated and employed with refugee populations, consequently limiting their validity. The questionnaires were assessed using interviews, which, to some extent, limits comparability with studies that used them as pen-and-paper self-reports. Data was primarily collected using interpreters, which may have introduced reporting biases; however, this can also be viewed as a strength in that it aided clarifications during the interviews. Furthermore, part of the study was conducted during the COVID-19 pandemic, and isolation due to pandemic restrictions can have acted as a confounder by exacerbating emotional distress among participants.

The study’s strengths include being the first to examine the validity of CPTSD in individuals with refugee backgrounds, incorporating children into the sample. Utilizing diagnostic interviews in addition to questionnaires, the study encompassed participants of all nationalities and included both accompanied and unaccompanied individuals. Additionally, the recruitment process was conducted nationwide.

### Conclusions

This study found a high prevalence of PTSD and CPTSD among a community sample of adolescents with refugee backgrounds residing in Sweden. The probable diagnostic rates for DSM-5 PTSD were significantly higher than ICD-11 PTSD and CPTSD. Distinct classes consistent with the ICD-11 formulation of PTSD and CPTSD were identified, and exposure to child maltreatment, along with exposure to more types of violence, were recognized as risk factors for CPTSD.

## Supplementary Information

Below is the link to the electronic supplementary material.

Supplementary file1 (DOCX 391 KB)

## Data Availability

The participants of this study did not give written consent for their data to be shared publicly, so supporting data is unavailable due to the sensitive nature of the research.

## Abbreviations

AIC: Akaike Information Criterion
aOR: Adjusted Odds Ratio
ARQ: Adolescent Resilience Questionnaire
BIC: Bayesian Information Criterion
BLRT: Bootstrapped Likelihood Ratio Test
CATS-1: Children and Adolescent Trauma Screen Version 1
CGAS: Children’s Global Assessment Scale
CI: Confidence Intervals
CPTSD: Complex Posttraumatic Stress Disorder
DSM-5: The Diagnostic and Statistical Manual of Mental Disorders, Fifth Edition
GAF: Global Assessment of Functioning
ICD-11: International Classification of Diseases 11th Revision
JVQ: Juvenile Victimization Questionnaire
LCA: Latent Class Analysis
MINI: Mini International Neuropsychiatric Interview
MINI-KID: Mini International Neuropsychiatric Interview for Children and Adolescents
OR: Odds Ratio
PCL-5: Posttraumatic Stress Disorder Checklist for DSM-5
PTE: Potentially Traumatic Event
PTSD: Posttraumatic Stress Disorder
PTSS: Posttraumatic Stress Symptoms
SD: Standard Deviation
WHO: World Health Organization
WHO-5: The World Health Organization- Five Well-Being Index

## Footnotes

1 Standardized scores.
