# Supplementary material for: Prevalence and discriminant validity of PTSD and CPTSD in a community sample of adolescents with refugee backgrounds residing in Sweden
Source: Eur Child Adolesc Psychiatry. 2025 Oct 8;35(2):575–86. doi: 10.1007/s00787-025-02858-8 (PMC12956912; doi:10.1007/s00787-025-02858-8)
Supplement: Supplementary file 1 — Supplementary file1 (DOCX 391 KB) [file 787_2025_2858_MOESM1_ESM.docx]

**Online Resource 1**

**Introduction**

Complete list of studies examining the factorial and discriminant validity of ICD-11 CPTSD in refugee samples (as of January 2025):

1. Tay AK, Rees S, Chen J, Kareth M, Silove D (2015) The structure of post-traumatic stress disorder and complex post-traumatic stress disorder amongst West Papuan refugees. BMC Psychiatry 15:111. <https://doi.org/10.1186/s12888-015-0480-3>

2. Nickerson A, Cloitre M, Bryant RA, Schnyder U, Morina N, Schick M (2016) The factor structure of complex posttraumatic stress disorder in traumatized refugees. European Journal of Psychotraumatology 7:33253. <https://doi.org/10.3402/ejpt.v7.33253>

3. Palic S, Zerach G, Shevlin M, Zeligman Z, Elklit A, Solomon Z (2016) Evidence of complex posttraumatic stress disorder (CPTSD) across populations with prolonged trauma of varying interpersonal intensity and ages of exposure. Psychiatry Research 246:692-699. <https://doi.org/10.1016/j.psychres.2016.10.062>

4. Silove D, Tay AK, Kareth M, Rees S (2017) The Relationship of Complex Post-traumatic Stress Disorder and Post-traumatic Stress Disorder in a Culturally Distinct, Conflict-Affected Population: A Study among West Papuan Refugees Displaced to Papua New Guinea. Frontiers in Psychiatry 8 <https://doi.org/10.3389/fpsyt.2017.00073>

5. Hyland P, Ceannt R, Daccache F, Abou Daher R, Sleiman J, Gilmore B, Byrne S, Shevlin M, et al. (2018) Are posttraumatic stress disorder (PTSD) and complex-PTSD distinguishable within a treatment-seeking sample of Syrian refugees living in Lebanon? Global Mental Health 5:e14. <https://doi.org/10.1017/gmh.2018.2>

6. Tay AK, Mohsin M, Rees S, Tam N, Kareth M, Silove D (2018) Factor structures of Complex Posttraumatic Stress Disorder and PTSD in a community sample of refugees from West Papua. Comprehensive Psychiatry 85:15-22. <https://doi.org/10.1016/j.comppsych.2018.05.001>

7. Vallières F, Ceannt R, Daccache F, Abou Daher R, Sleiman J, Gilmore B, Byrne S, Shevlin M, et al. (2018) ICD‐11 PTSD and complex PTSD amongst Syrian refugees in Lebanon: the factor structure and the clinical utility of the International Trauma Questionnaire. Acta Psychiatrica Scandinavica 138:547-557. <https://doi.org/10.1111/acps.12973>

8. Barbieri A, Visco-Comandini F, Alunni Fegatelli D, Schepisi C, Russo V, Calò F, Dessì A, Cannella G, et al. (2019) Complex trauma, PTSD and complex PTSD in African refugees. European Journal of Psychotraumatology 10:1700621. <https://doi.org/10.1080/20008198.2019.1700621>

9. Frost R, Hyland P, McCarthy A, Halpin R, Shevlin M, Murphy J (2019) The complexity of trauma exposure and response: Profiling PTSD and CPTSD among a refugee sample. Psychological Trauma: Theory, Research, Practice &amp; Policy 11:165-175. <https://doi.org/10.1037/tra0000408>

10. Liddell BJ, Nickerson A, Felmingham KL, Malhi GS, Cheung J, Den M, Askovic M, Coello M, et al. (2019) Complex Posttraumatic Stress Disorder Symptom Profiles in Traumatized Refugees. Journal of Traumatic Stress 32:822-832. <https://doi.org/10.1002/jts.22453>

11. Vang ML, Nielsen SB, Auning-Hansen M, Elklit A (2019) Testing the validity of ICD-11 PTSD and CPTSD among refugees in treatment using latent class analysis. Torture 29:27-45. <https://doi.org/10.7146/torture.v29i3.115367>

12. Vang ML, Dokkedahl SB, Løkkegaard SS, Jakobsen AV, Møller L, Auning-Hansen MA, Elklit A (2021) Validation of ICD-11 PTSD and DSO using the International Trauma Questionnaire in five clinical samples recruited in Denmark. European Journal of Psychotraumatology 12:1894806. <https://doi.org/10.1080/20008198.2021.1894806>

13. Baek J, Kim K-A, Kim H, Kim O, Ko M, Kim SH, Sohn IB, Shin BK, et al. (2022) The validity of ICD-11 PTSD and complex PTSD in North Korean defectors using the International Trauma Questionnaire. European Journal of Psychotraumatology 13:2119012. <https://doi.org/10.1080/20008066.2022.2119012>

14. Barbieri A, Saidou Soumana S, Dessì A, Sadou O, Boubacar T, Visco-Comandini F, Alunni Fegatelli D, Pirchio S (2023) Complex PTSD in asylum-seekers living in a humanitarian setting in Africa: A latent class analysis. Psychological Trauma 15:1136-1144. <https://doi.org/10.1037/tra0001299>

**Methods**

***Procedure***

Regarding transformation of variables, country of origin was transformed into region of origin (North Africa and the Middle East, Sub-Saharan Africa, and Other) for confidentiality purposes and parental education was coded into low, medium, and high categories based on the Swedish standard education classification [1]. Education level for mother and father was then combined into a single variable based on the highest education level of either parent.

***Measures***

The Juvenile Victimization Questionnaire (JVQ) was tailored for the larger study by incorporating common refugee experiences related to natural disasters, accidents, medical procedures, the death of close relatives, poverty, separation from their parents, being captured or imprisoned, and human trafficking. The questionnaire has been extensively validated and used in studies measuring exposure to violence among children and adolescents in a range of countries, including the United Kingdom, the United States, China, Switzerland and Pakistan [2-4]. It has also been used in studies with samples which has included young adults [5, 6]. The JVQ has not yet been validated or extensively utilized in populations with refugee backgrounds. The following questions were added to the questionnaire for this study.

- Have you experienced a serious natural disaster such as a flood, tornado, hurricane, earthquake or fire?
- Serious accident or injury such as, car/bike accident, dog bite or sports injury?
- Stressful or frightening medical examination?
- Sudden or violent death of someone close to you?
- Growing up, have you experienced poverty in your family?
- Have you had to work to support yourself or your family?
- Have you had to be separated from your parents or someone important to you because of war or other disturbances?
- Have you been captured or imprisoned in any way?
- Have you been involved in human trafficking for sexual purposes?
- Have you been trafficked for the removal of organs?
- Have you been trafficked for military service?
- Have you been trafficked for forced labour (i.e. you have been forced to work without pay or for very little pay. This also includes domestic work, if it was more extensive than is usual for someone of your age)?
- Have you been trafficked for any other purpose, in a situation of distress for the victim, e.g. begging or committing a crime? Or something else similar that we have not asked about? To interviewer: if so, please write this in the comment section.

The Child and Adolescent Trauma Screen version 1 (CATS-1) has previously been used in German study with a population of children and adolescents with refugee backgrounds (α=0.81) [7]. Additionally, its validity and reliability has been tested across several languages and accompanying populations [8].

The Posttraumatic Stress Disorder Checklist for DSM-5 (PCL-5) has previously been used in a study on refugees in Germany (α=0.93-0.97) [9], and more recently among displaced Palestinian young adults from Gaza (α=0.92) [10], and Syrian refugee women in Jordan (α=0.94) [11].

The WHO-5 has previously been used in a study investigating mental illness in Syrian refugees resettled in Sweden (α=0.94) [12]. The questionnaire was also used in a study on refugees resettled in Denmark [13], and recently in a study on adolescent girls in Palestinian refugee camps (α=0.81) [14].

The Adolescent Resilience Questionnaire (ARQ) has been translated into and tested in multiple different languages, including Swedish [15]. However, it has yet to be validated in a sample of children and adolescents with refugee backgrounds and has mainly been used with adolescents under the age of 19. For the larger study, we selected the following items from the ARQ. The highest loading item for each subscale (Self, Family, Peers, School and Society) based on previous studies [15, 16] was selected for use in the interview.

ARQ item Self:
*I feel confident that I can handle whatever comes my way.*

ARQ item Family:
*I enjoy spending time with my family.*

ARQ item Peers:
*I have a friend I can trust with my private thoughts and feelings.*

ARQ item School
*My teachers are caring and supportive of me.*

ARQ item Society
*I trust people in my neighborhood.*

The Mini International Neuropsychiatric Interview for Children and Adolescents 6.0 (MINI-KID) has been validated in Sweden [17] and previously been used and rigorously tested in a population of Syrians with refugee backgrounds residing in Lebanon [18]. The Mini International Neuropsychiatric Interview 7.0 (MINI) has previously been used in several different refugee populations [19, 20].

***Reliability***

Internal consistency:

WHO-5

α = 0.86

CATS-1

Total scale: α = 0.94

Cluster B: α = 0.87

Cluster C: α = 0.72

Cluster D: α = 0.84

Cluster E: α = 0.80

PCL-5

Total scale: α = 0.90

Cluster B: α = 0.75

Cluster C: α = 0.67

Cluster D: α = 0.77

Cluster E: α = 0.80

**Table S1. Item selection from CATS-1 and PCL-5 for use with ICD-11 criteria.**

| **ICD-11 PTSD CRITERIA** | **PCL-5 & CATS-1 ITEMS** |
| --- | --- |
| **Re-experiencing** | **Re-experiencing** |
| Re-experiencing the traumatic event after the traumatic event has occurred, in which the event(s) is not just remembered but is experienced as occurring again in the here and now. This typically occurs in the form of vivid intrusive memories or images; flashbacks, which can vary from mild (there is a transient sense of the event occurring again in the present) to severe (there is a complete loss of awareness of present surroundings), or repetitive dreams or nightmares that are thematically related to the traumatic event(s). Re-experiencing is typically accompanied by strong or overwhelming emotions, such as fear or horror, and strong physical sensations. Re-experiencing in the present can also involve feelings of being overwhelmed or immersed in the same intense emotions that were experienced during the traumatic event, without a prominent cognitive aspect, and may occur in response to reminders of the event. Reflecting on or ruminating about the event(s) and remembering the feelings that one experienced at that time are not sufficient to meet the re-experiencing requirement. | PCL-5 [2]: Repeated, disturbing dreams of the stressful experience?  PCL-5 [3]: Suddenly feeling or acting as if the stressful experience were actually happening again (as if you were actually back there reliving it)?  CATS-1 [2]: Bad dreams reminding you of what happened.  CATS-1 [3]: Feeling as if what happened is happening all over again. |
| **Avoidance** | **Avoidance** |
| Deliberate avoidance of reminders likely to produce re-experiencing of the traumatic event(s). This may take the form either of active internal avoidance of thoughts and memories related to the event(s), or external avoidance of people, conversations, activities, or situations reminiscent of the event(s). In extreme cases the person may change their environment (e.g., move house or change jobs) to avoid reminders. | PCL-5 [6]: Avoiding memories, thoughts, or feelings related to the stressful experience?  PCL-5 [7]: Avoiding external reminders of the stressful experience (for example, people, places, conversations, activities, objects, or situations)?  CATS-1 [6]: Trying not to think about what happened. Or to not have feelings about it.  CATS-1 [7]: Staying away from anything that reminds you of what happened (people, places, things, situations, talks). |
| **Sense of current threat** | **Sense of current threat** |
| Persistent perceptions of heightened current threat, for example as indicated by hypervigilance or an enhanced startle reaction to stimuli such as unexpected noises. Hypervigilant persons constantly guard themselves against danger and feel themselves or others close to them to be under immediate threat either in specific situations or more generally. They may adopt new behaviours designed to ensure safety (not sitting with ones’ back to the door, repeated checking in vehicles’ rear-view mirror). In Complex Post-Traumatic Stress Disorder, unlike in Post-Traumatic Stress Disorder, the startle reaction may in some cases be diminished rather than enhanced. | PCL-5 [17]: Being “superalert” or watchful or on guard?  PCL-5 [18]: Feeling jumpy or easily startled?  CATS-1 [17]: Being overly careful (checking to see who is around you).  CATS-1 [18]: Being jumpy. |
| **ICD-11 DSO CRITERIA** | **PCL-5 & CATS-1 ITEMS** |
| **Affect dysregulation** | **Affect dysregulation** |
| Severe and pervasive problems in affect regulation. Examples include heightened emotional reactivity to minor stressors, violent outbursts, reckless or self-destructive behaviour, dissociative symptoms when under stress, and emotional numbing, particularly the inability to experience pleasure or positive emotions. | PCL-5 [14]: Trouble experiencing positive feelings (for example, being unable to feel happiness or have loving feelings for people close to you)?  PCL-5 [15]: Irritable behavior, angry outbursts, or acting aggressively?  CATS-1 [14]: Not being able to have good or happy feelings.  CATS-1 [15]: Feeling mad. Having fits of anger and taking it out on others. |
| **Negative self-concept** | **Negative self-concept** |
| Persistent beliefs about oneself as diminished, defeated or worthless, accompanied by deep and pervasive feelings of shame, guilt or failure related to the stressor. For example, the individual may feel guilty about not having escaped from or succumbing to the adverse circumstance, or not having been able to prevent the suffering of others. | PCL-5 [9]: Having strong negative beliefs about yourself, other people, or the world (for example, having thoughts such as: I am bad, there is something seriously wrong with me, no one can be trusted, the world is completely dangerous)?  PCL-5 [10]: Blaming yourself or someone else for the stressful experience or what happened after it?  CATS-1 [9]: Negative thoughts about yourself or others. Thoughts like I won’t have a good life, no one can be trusted, the whole world is unsafe.  CATS-1 [10]: Blaming yourself for what happened. Or blaming someone else when it isn’t their fault. |
| **Disturbed relation** | **Disturbed relation** |
| Persistent difficulties in sustaining relationships and in feeling close to others. The person may consistently avoid, deride or have little interest in relationships and social engagement more generally. Alternatively, there may be occasional intense relationships, but the person has difficulty sustaining them. | PCL-5 [13]: Feeling distant or cut off from other people?  CATS-1 [13]: Not feeling close to people.  ARQ [3]: I have a close friend with whom I can share my private thoughts and feelings. |

**Table S2. Symptom cluster endorsement per class.**

| **Cluster** | **Class 1 Low symptoms, N (%)** | **Class 2 CPTSD, N (%)** | **Class 3 PTSD, N (%)** |
| --- | --- | --- | --- |
| Re-experiencing | 5 (4.1%) | 56 (86.2%) | 24 (33.8%) |
| Avoidance | 8 (6.6%) | 62 (95.4%) | 54 (76.1%) |
| Sense of threat | 21 (17.2%) | 58 (89.2%) | 48 (67.6%) |
| Affect dysregulation | 1 (0.8%) | 65 (100%) | 26 (36.6%) |
| Negative self-concept | 16 (13.1%) | 60 (92.3%) | 40 (56.3%) |
| Disturbed relation | 12 (9.8%) | 57 (87.7%) | 35 (49.3%) |

**Figure S1. Profile plot for 2 classes.**

**
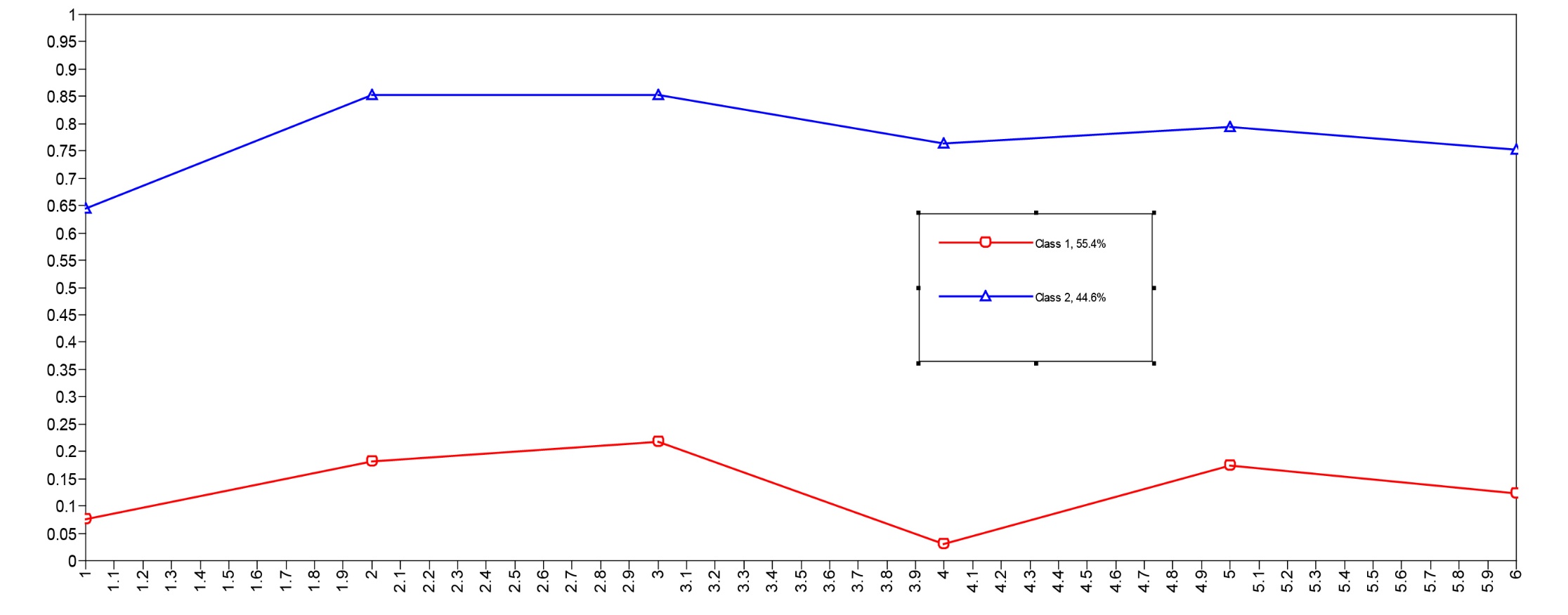
**

**Table S3. Descriptives of covariates of classes and differences between CPTSD and PTSD based on unadjusted regression model with additional variables.**

| **Covariates** | **Total LCA sample**  **(n = 258)** | **Class 1 Low symptoms**  **(n = 122)** | **Class 2 CPTSD (n = 65)** | **Class 3 PTSD**  **(n = 71)** | **Significant differences between CPTSD and PTSD**  **(p < 0.05)** |
| --- | --- | --- | --- | --- | --- |
| Age, years, mean (SD^a^)  Min/max  Missing | 18.21 (3.75)  12/25  0 | 16.33 (3.17)  12/25  0 | 19.98 (3.33)  12/25  0 | 19.83 (3.50)  12/25  0 |  |
| Gender, female, n (%)  Missing | 115 (44.6%)  0 | 58 (47.5%)  0 | 26 (40.0%)  0 | 31 (43.7%)  0 |  |
| Unaccompanied status, n (%)  Missing | 61 (23.6%)  0 | 11 (9.0%)  0 | 29 (44.6%)  0 | 21 (29.6%)  0 |  |
| Exposure to number of different types of violence, mean (SD)  Min/max  Missing | 10.97 (7.22)  0/34  1 | 7.33 (6.03)  0/25  1 | 15.78 (7.10)  4/34  0 | 12.76 (5.88)  2/25  0 | CPTSD > PTSD |
| Exposure to sexual victimization, yes, n (%)  Missing | 66 (25.8%)  2 | 15 (12.4%)  1 | 29 (44.6%)  0 | 22 (31.4%)  1 |  |
| Exposure to child maltreatment, yes, n (%)  Missing | 120 (46.9%)  2 | 34 (28.1%)  1 | 47 (72.3%)  0 | 39 (55.7%)  1 | CPTSD > PTSD |
| CATS-1, mean (SD)  Min/max  Missing | 11.77 (13.07)  0/54  0 | 5.20 (5.70)  0/19  0 | 38.23 (7.03)  28.50/54  0 | 22.58 (8.39)  8/36  0 | See PTSS |
| PCL-5, mean (SD)  Min/max  Missing | 29.91 (17.09)  0/69  0 | 10.84 (6.05)  0/23  0 | 45.69 (11.30)  22/69  0 | 24.43 (10.90)  8/57.89  0 | See PTSS |
| PTSS, mean^b^ (SD)  Min/max  Missing | See CATS-1 and PCL-5 | -0.82 (0.49)  -1.75/0.55  0 | 1.18 (0.79)  -0.46/3.23  0 | 0.02 (0.83)  -1.28/1.85  0 | CPTSD > PTSD |
| GAF/C-GAS, mean (SD)  Median  Min/max  Missing | 80.65 (14.64)  85  31/100  4 | 86.63 (9.47)  90  55/100  3 | 68.08 (15.70)  69.50  35/98  1 | 81.96 (13.90)  86  31/97  0 | CPTSD < PTSD |
| WHO-5, mean (SD)  Median  Min/max  Missing | 69.56 (24.59)  72  0/100  9 | 80.58 (20.50)  88  20/100  5 | 52.00 (25.40)  52  0/100  4 | 65.97 (20.60)  68  12/100  0 | CPTSD < PTSD |
| Suicide attempts, yes, n (%)  Missing | 7 (3.2%)  38 | 0 (0%)  23 | 6 (10.52%)  8 | 1 (1.6%)  7 | Sample to small |
| Suicide thoughts, yes, n (%)  Missing | 33 (14.2%)  26 | 6 (5.9%)  20 | 19 (30.6%)  3 | 8 (11.8%)  3 | CPTSD > PTSD |
| NSSI, yes, n (%)  Missing | 17 (7.6%)  33 | 3 (3.0%)  21 | 10 (16.9%)  6 | 4 (6.2%)  6 | Sample to small |
| Any other diagnosis, yes, n (%)  Missing | 71 (28.1%)  5 | 16 (13.4%)  3 | 40 (62.5%)  1 | 15 (21.4%)  1 | CPTSD > PTSD |
| Sought treatment, yes, n (%)  Missing | 57 (22.7%)  7 | 13 (10.9%)  3 | 28 (45.2%)  3 | 16 (22.9%)  1 | CPTSD > PTSD |

^a^, Standard deviation

^b^, Standardized scores

**Figure S2. Profile plot for 3 classes with an additional item added to the re-experience cluster.**


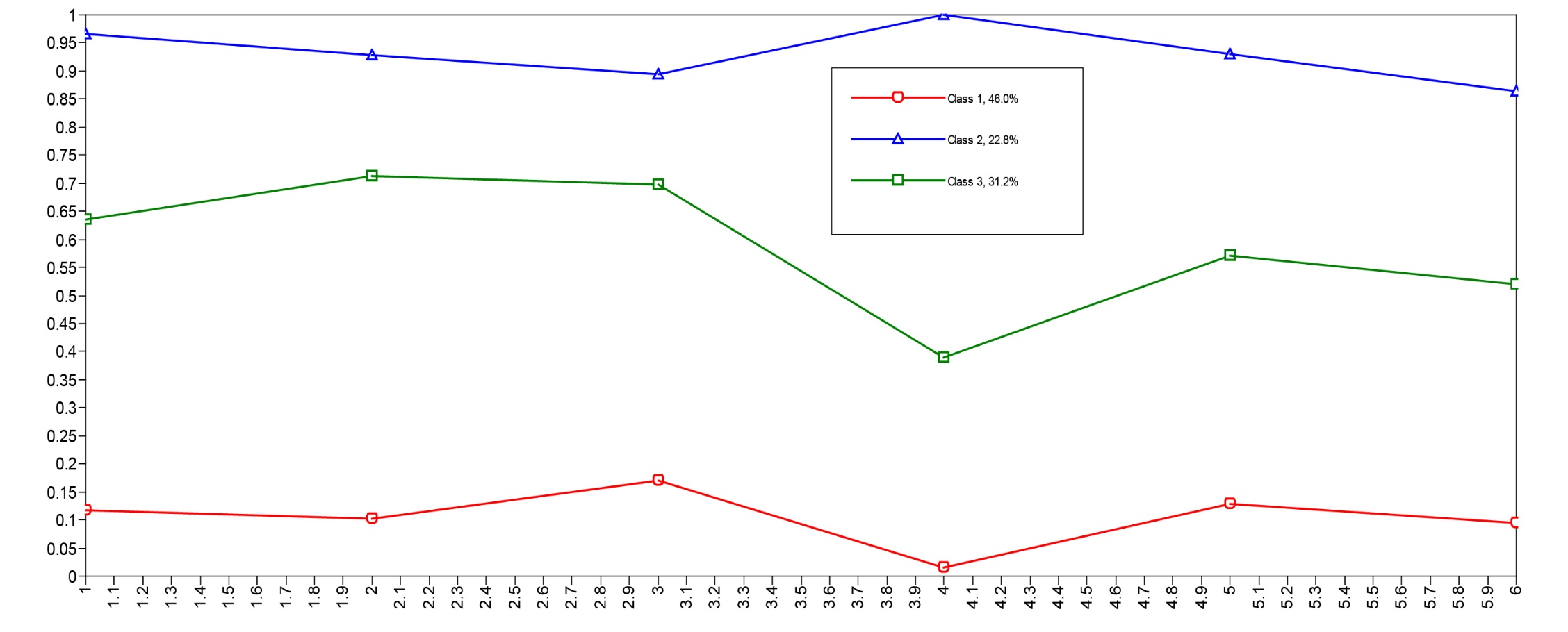


**References**

1. Statistics Sweden (2020) Swedish Standard Classification of Education. <https://www.scb.se/dokumentation/klassifikationer-och-standarder/svensk-utbildningsnomenklatur-sun/>.

2. Haahr-Pedersen I, Ershadi AE, Hyland P, Hansen M, Perera C, Sheaf G, Bramsen RH, Spitz P, et al. (2020) Polyvictimization and psychopathology among children and adolescents: A systematic review of studies using the Juvenile Victimization Questionnaire. Child Abuse Negl. 107:104589. <https://doi.org/10.1016/j.chiabu.2020.104589>

3. Meinck F, Neelakantan L, Steele B, Jochim J, Davies LM, Boyes M, Barlow J, Dunne M (2023) Measuring Violence Against Children: A COSMIN Systematic Review of the Psychometric Properties of Child and Adolescent Self-Report Measures. Trauma Violence Abus 24:1832-1847. <https://doi.org/10.1177/15248380221082152>

4. Mathews B, Pacella R, Dunne MP, Simunovic M, Marston C (2020) Improving measurement of child abuse and neglect: A systematic review and analysis of national prevalence studies. PLoS One 15:e0227884. <https://doi.org/10.1371/journal.pone.0227884>

5. Pinto-Cortez C, Gutiérrez-Echegoyen P, Henríquez D (2021) Child Victimization and Polyvictimization Among Young Adults in Northern Chile. J Interpers Violence 36:2008-2030. <https://doi.org/10.1177/0886260518759058>

6. Emmerich OLM, Wagner B, Heinrichs N, van Noort BM (2024) Lifetime victimization experiences, depressiveness, suicidality, and feelings of loneliness in youth in care. Child Abuse Negl. 154:106870. <https://doi.org/10.1016/j.chiabu.2024.106870>

7. Müller LRF, Gossmann K, Schmid RF, Rosner R, Unterhitzenberger J (2021) A pilot study on ecological momentary assessment in asylum-seeking children and adolescents resettled to Germany: Investigating compliance, post-migration factors, and the relation between daily mood, sleep patterns, and mental health. PLoS One 16:e0246069. <https://doi.org/10.1371/journal.pone.0246069>

8. Sachser C, Berliner L, Holt T, Jensen TK, Jungbluth N, Risch E, Rosner R, Goldbeck L (2017) International development and psychometric properties of the Child and Adolescent Trauma Screen (CATS). J. Affect. Disord. 210:189-195. <https://doi.org/10.1016/j.jad.2016.12.040>

9. Nesterko Y, Jäckle D, Friedrich M, Holzapfel L, Glaesmer H (2020) Prevalence of post-traumatic stress disorder, depression and somatisation in recently arrived refugees in Germany: an epidemiological study. Epidemiol. Psychiatr. Sci. 29:e40. <https://doi.org/10.1017/S2045796019000325>

10. Aldabbour B, Abuabada A, Lahlouh A, Halimy M, Elamassie S, Sammour AA-K, Skaik A, Nadarajah S (2024) Psychological impacts of the Gaza war on Palestinian young adults: a cross-sectional study of depression, anxiety, stress, and PTSD symptoms. BMC Psychology 12:696. <https://doi.org/10.1186/s40359-024-02188-5>

11. Brooks MA, Dasgupta A, Khadra M, Bawaneh A, Kaushal N, El-Bassel N (2024) Suicidal behaviors among refugee women in Jordan: post-traumatic stress disorder, social support and post-displacement stressors. BMC Public Health 24:2677. <https://doi.org/10.1186/s12889-024-20128-1>

12. Tinghög P, Malm A, Arwidson C, Sigvardsdotter E, Lundin A, Saboonchi F (2017) Prevalence of mental ill health, traumas and postmigration stress among refugees from Syria resettled in Sweden after 2011: a population-based survey. BMJ Open 7:e018899. <https://doi.org/10.1136/bmjopen-2017-018899>

13. Sander R, Laugesen H, Skammeritz S, Mortensen EL, Carlsson J (2019) Interpreter-mediated psychotherapy with trauma-affected refugees – A retrospective cohort study. Psychiatry Res. 271:684-692. <https://doi.org/10.1016/j.psychres.2018.12.058>

14. Ghandour R, Hammoudeh W, Stigum H, Giacaman R, Fjeld H, Holmboe-Ottesen G (2024) The hidden burden of dysmenorrhea among adolescent girls in Palestine refugee camps: a focus on well-being and academic performance. BMC Public Health 24:726. <https://doi.org/10.1186/s12889-024-18219-0>

15. Nilsson D, Svedin CG, Hall F, Kazemi E, Dahlström Ö (2022) Psychometric properties of the Adolescent Resilience Questionnaire (ARQ) in a sample of Swedish adolescents. BMC Psychiatry 22 <https://doi.org/10.1186/s12888-022-04099-4>

16. Gartland D, Bond L, Olsson CA, Buzwell S, Sawyer SM (2011) Development of a multi-dimensional measure of resilience in adolescents: the Adolescent Resilience Questionnaire. BMC Med. Res. Methodol. 11:134. <https://doi.org/10.1186/1471-2288-11-134>

17. Högberg C, Billstedt E, Björck C, Björck P-O, Ehlers S, Gustle L-H, Hellner C, Höök H, et al. (2019) Diagnostic validity of the MINI-KID disorder classifications in specialized child and adolescent psychiatric outpatient clinics in Sweden. BMC Psychiatry 19:142. <https://doi.org/10.1186/s12888-019-2121-8>

18. Kyrillos V, Bosqui T, Moghames P, Chehade N, Saad S, Abdul Rahman D, Karam E, Karam G, et al. (2023) The culturally and contextually sensitive assessment of mental health using a structured diagnostic interview (MINI Kid) for Syrian refugee children and adolescents in Lebanon: Challenges and solutions. Transcult Psychiatry 60:125-141. <https://doi.org/10.1177/13634615221105114>

19. Hocking DC, Mancuso SG, Sundram S (2018) Development and validation of a mental health screening tool for asylum-seekers and refugees: the STAR-MH. BMC Psychiatry 18:69. <https://doi.org/10.1186/s12888-018-1660-8>

20. Bogic M, Njoku A, Priebe S (2015) Long-term mental health of war-refugees: a systematic literature review. BMC Int. Health Hum. Rights 15 <https://doi.org/10.1186/s12914-015-0064-9>
